# Cholecystokinin Expression in the Development of Myocardial Hypertrophy

**DOI:** 10.1155/2021/8231559

**Published:** 2021-08-21

**Authors:** Zhongshu Han, Sheng Bi, Yongsheng Xu, Xiaoying Dong, Lixia Mei, Hailong Lin, Xueqi Li

**Affiliations:** ^1^Department of Cardiology, Harbin Medical University Fourth Hospital, Harbin 150086, China; ^2^Department of Critical Care Medicine, Affiliated Qiqihar Hospital, Southern Medical University (The First Hospital of Qiqihar), Qiqihar 161005, China; ^3^Department of Anesthesiology, Affiliated Hospital of Qingdao University, Qingdao 266000, China; ^4^Department of Ultrasonic Medicine, Affiliated Qiqihar Hospital, Southern Medical University (The First Hospital of Qiqihar), Qiqihar 161005, China; ^5^Department of Cardiology, Central Hospital of Dalian, Dalian 116003, China

## Abstract

**Background:**

Expression of cholecystokinin is found in myocardial tissues as a gastrointestinal hormone and may be involved in cardiovascular regulation. However, it is unclear whether there is an increase in cholecystokinin expression in myocardial hypertrophy progression induced by abdominal aortic constriction. The study is aimed at exploring the relationship between cholecystokinin expression and myocardial hypertrophy.

**Methods:**

We randomly divided the 70 Sprague-Dawley rats into two groups: the sham operation group and the abdominal aortic constriction group. The hearts of rats were measured by echocardiography, and myocardial tissues and blood were collected at 4 weeks, 8 weeks, and 12 weeks after surgery. Morphological changes were assessed by microscopy. The cholecystokinin expression was evaluated by immunochemistry, Western blotting, quantitative real-time polymerase chain reaction, and enzyme-linked immunosorbent assay.

**Results:**

The relative protein levels of cholecystokinin were significantly increased in the abdominal aortic constriction groups compared with the corresponding sham operation groups at 8 weeks and 12 weeks. The cholecystokinin mRNA in the abdominal aortic constriction groups was significantly higher than the time-matched sham operation groups. Changes in the left ventricular wall thickness were positively correlated with the relative protein levels of cholecystokinin and the mRNA of cholecystokinin.

**Conclusions:**

The development of myocardial hypertrophy can affect the cholecystokinin expression of myocardial tissues.

## 1. Introduction

The heart is not only a contractile organ but also an endocrine organ. There is evidence that the cardiomyocytes produce a variety of peptide hormones, such as the natriuretic peptides [1], parathyroid hormone-like protein [2], endothelin [3], and relaxin [4]. These hormones affect the function of the cardiovascular system. A recent study has found that gastrointestinal hormones are associated with the change of the heart's structure and function [5], and CCK is one of these gastrointestinal hormones. It is made up of 33 amino acids, and all biological activity is present in the C-terminal octapeptide fragment. It initially extracts from the upper segment of the intestine of the gut [6]. The study showed that cardiomyocytes could produce the classic intestinal hormone cholecystokinin (CCK) in amounts comparable to natriuretic prohormones, and CCK was found in the lung, kidney, gastrointestinal tract, and brain later [7].

The abnormal cardiomyocytes can induce the expression of CCK. The study found that CCK expression in the postinfarction heart failure (HF) group was higher than that in the sham group. So, HF can induce the expression of CCK [8]. Whether hypoxia caused by myocardial infarction can affect the expression of CCK? The study reported that acute myocardial hypoxia increases the BNP mRNA contents in porcine heart muscle [9]. However, using the myocardial hypoxia model, there was no significant different in the expression of pro-CCK between the myocardial hypoxia groups and the control groups, so myocardial hypoxia does not affect the expression of CCK in myocardial tissues [10]. From the above description, we know that HF can lead to high CCK expression, the feature is similar to an increase in the BNP caused by HF. The part of HF develops from myocardial hypertrophy (MH) caused by the high-pressure load of the heart. The expression of CCK in MH tissue is not clear.

In this study, we applied a rat model of (4-12 weeks) abdominal aortic constriction- (AAC-) related cardiac pressure overload with morphological changes of MH, to systematically investigate CCK expression in the MH tissue.

## 2. Methods

The study was performed in accordance with the Guide for the Care and Use of Laboratory Animals published by the US National Institutes of Health (NIH Publication No. 85-23, revised 19-96). The experiment protocols were approved by the Research Ethics Committee of Harbin Medical University Fourth Hospital.

### 2.1. Rat Model of MH

70 male Sprague-Dawley rats (weighing 310-330 g, 11-13 weeks of age) were purchased from the Animal Experimental Center of the Second Affiliated Hospital of Harbin Medical University; the number of rats was determined by preliminary experiment. Rats were free to eat and drink. 4 rats were put in a cage with the room temperature at 22°C and room humidity 50 ± 5%, maintained on 12 hours light/dark cycles for 7 days before surgery.

We made a rat model of MH by abdominal aortic constriction operation as described previously [11] with a few modifications. The animals were randomly divided into two groups: (1) the sham operation (sham) group (*n* = 30), in which the surgical procedure of AAC was performed in the same way except for ligation of the abdominal aorta, and (2) the AAC group (*n* = 40), in which the surgical procedure of AAC was performed by ligation of the abdominal aorta 5 mm above the branching of the right renal artery to induce pressure overload of the heart. The rats were well anesthetized with 4% pentobarbital sodium (40 mg/kg, intraperitoneal injection) and placed on the operating table. Skin preparation and iodine disinfection were carried out on the abdomen of the rats. An incision of about 2.0 cm was made on midline's left side, and the abdominal cavity was opened layer by layer. The intestines were pushed to the right side of the abdominal cavity, while the stomach was pushed upwards to expose the abdominal aorta (5 mm above the right renal artery branches). A needle (0.7 mm in diameter) was laid alongside the abdominal aorta, and they were bundled together with a surgical suture (0.3 mm in diameter); the needle was taken out. Internal organs were restored, and penicillin (200,000 U) was infused into the abdominal cavity. The abdominal wall was sutured layer by layer. The rats were kept warm until they were awake. During the next 7 days, the rats were given penicillin (200,000 U/day) by intraperitoneal injection. All surgeries were performed using sterile techniques. For the sham group, in addition to the abdominal aorta was not bundled, other steps are the same. 12 rats died of surgery. Surviving rats of the sham group were randomly assigned into 4 weeks sham group (9 rats), 8 weeks sham group (9 rats), and 12 weeks sham group (10 rats); surviving rats of the AAC group were randomly assigned into 4 weeks AAC group (10 rats), 8 weeks AAC group (10 rats), and 12 weeks AAC group (10 rats).

### 2.2. Echocardiography

The rats were anesthetized and prepared skin and applied an ultrasonic coupling agent to the abdomen. The systolic cardiac function and the dimension of heart's left ventricular were measured 4 weeks, 8 weeks, and 12 weeks after surgery by two-dimensional echocardiography (SONOS 7500, Philips) fitted with a 12-MHz transducer. The operator of an ultrasonic machine was blinded to the sham and ACC groups. Data were recorded as the mean of at least three consecutive cardiac cycles. The parameters recorded included left atrial diameter (LA), interventricular septal thickness at end-diastole (IVSd), left ventricular end-diastolic posterior wall thickness (LVPWd), left ventricular end-systolic diameter (LVDs), left ventricular end-diastolic diameter (LVDd), left ventricular fractional shortening (LVFS), and the left ventricular ejection fraction (LVEF).

### 2.3. The Blood Sample Collection

The blood samples of rats from each group were obtained from the carotid artery on the operating table after rats were well anesthetized with 4% pentobarbital sodium (40 mg/kg, intraperitoneal injection). Blood samples were centrifuged at 3000 x g for 15 min, and then, the plasma was collected for measurement and stored at -20°C.

### 2.4. Procedure of Euthanasia

After euthanasia using an overdose of pentobarbital, the breathing and heartbeat of the rats stopped, and the pupils dilated.

### 2.5. The Myocardial Tissue Sample Collection

The hearts from rats of sham groups and ACC groups were excised and irrigated with cold saline solution after rats were euthanized. Each heart weight (HW) was obtained by high-accuracy electronic scale. Subsequently, the ratio of heart weight to body weight (HW/BW) was calculated. A portion of the myocardial tissues was fixed in 4% paraformaldehyde for histological analysis, another portion was stored in liquid nitrogen for Western blotting (WB), and the remaining myocardial samples were saturated in RNA later Stabilization Solution (Thermo Fisher Scientific, Massachusetts, USA) and stored at -80°C.

### 2.6. Morphological Changes in the Myocardium

Myocardial tissue samples were fixed in formaldehyde and embedded in paraffin. After that, they were cut into 5 *μ*m thick sections and stained with hematoxylin and eosin (H&E) to analyze myocardial morphology and Masson's trichrome to evaluate the severity of myocardial fibrosis. Their pathological slides were randomly selected from three rats in each group for myocardial morphological analysis.

### 2.7. Quantitative Real-Time PCR for CCK Expression Measurements

CCK mRNA expression levels in the left ventricle myocardial tissue were detected by quantitative real-time polymerase chain reaction (qPCR). RNA was extracted from myocardial tissues using a high pure RNA isolation kit (Roche, Basel, Switzerland) according to manufacturer's protocol. Transcriptor Reverse Transcriptase (Roche, Basel, Switzerland) and Protector RNase Inhibitor were used to synthesize cDNA. Fast Start Universal SYBR Green Master (Roche, Basel, Switzerland), cDNA templates, and specific primers were mixed, and amplification was performed using Step One Plus™ PCR system (Applied Biosystems, Foster, CA, USA). The sequences of the primer were listed in Table 1. GAPDH was used as an internal standard. The program was comprised of 95°C for 10 min, followed by 40 cycles at 95°C for 15 s, and 60°C for 1 min. The relative expression levels of CCK were determined using the 2^–△△Ct^ method.

### 2.8. Immunohistochemistry (IHC) for CCK Expression Measurements

The myocardial tissue samples were fixed with 4% paraformaldehyde, embedded in paraffin, and then, sectioned at a thickness of 4 *μ*m. The sections were subsequently deparaffinized and rehydrated, after that they were incubated with a rabbit anti-rat polyclonal antibody against CCK (EterLife, Birmingham, UK) diluted 1 : 100 overnight at 4°C. The sections were then counterstained with hematoxylin and observed under the microscope (Leica DM4B, Germany).

### 2.9. WB for CCK Expression Measurements

To measure endogenous CCK expression levels, myocardial tissues were ground and homogenized with a protein lysis solution (Sclarbio, China). Briefly, the frozen myocardium stored in liquid nitrogen was homogenized in RIPA lysis buffer (RIPA) with a protease inhibitor cocktail (Roche, South San Francisco, CA, USA) using a tissue grinder. The mixture was centrifuged at 12,000 rpm for 15 min at 4°C. Supernatants were obtained and assayed for total protein using the BCA method (Beyotime biotechnology, China). Equivalent amounts of protein were resolved on 15% Trisglycine sodium dodecyl sulphate (SDS) polyacrylamide gels and transferred to nitrocellulose membranes. After blocking in 5% dried milk in Tris-buffered saline containing Tween-20 for 1 h at room temperature, membranes were incubated for 24 h at 4°C with one of the following antibodies: anti-CCK polyclonal antibody (Santa Cruz Biotechnology, USA) and anti-GAPDH (ZSGB-BIO, China) to obtain the proteins levels. Membranes were incubated with horseradish peroxidase-conjugated donkey anti-rabbit or anti-mouse immunoglobulin (1 : 5,000; ZSGB-BIO, China) for 1 h at room temperature. All images were captured using the enhanced chemiluminescence Western blotting detection system (ChemiDoc XRS+, Bio-Rad, USA) and analyzed by densitometry. The optical density values were normalized to that of GAPDH.

### 2.10. Enzyme-Linked Immunosorbent Assay (ELISA) for CCK Expression Measurements

Plasma CCK levels were obtained by using enzyme-linked immunoassay kits (#JL11700, Jianglai Biological, China), according to manufacturer's protocol. The lower limit of detection for CCK was 1.0 pg/ml.

### 2.11. Statistical Analysis

Statistical analysis software was IBM SPSS 23 Statistics (IBM, Armonk, New York, USA). Data were assessed for normality using the Kolmogorov-Smirnov test. The results are presented as mean values ± standard deviation (SD). Statistical analysis of sham groups and AAC groups were performed using independent samples *t*-tests or Mann-Whitney test. Statistical analysis of three different groups at three-time points in AAC groups was performed using ANOVA or the Kruskal-Wallish; multiple comparison tests were performed using Mann-Whitney *U* or SNK-q (*a* = 0.05). Spearman's correlation coefficient was applied to study the associations between CCK expression and left ventricular wall thickness. All tests were two-sided, and the statistical significance was defined as *P* < 0.05.

## 3. Results

### 3.1. Body Weight and Cardiac Structural Parameters

**Figure 1:**
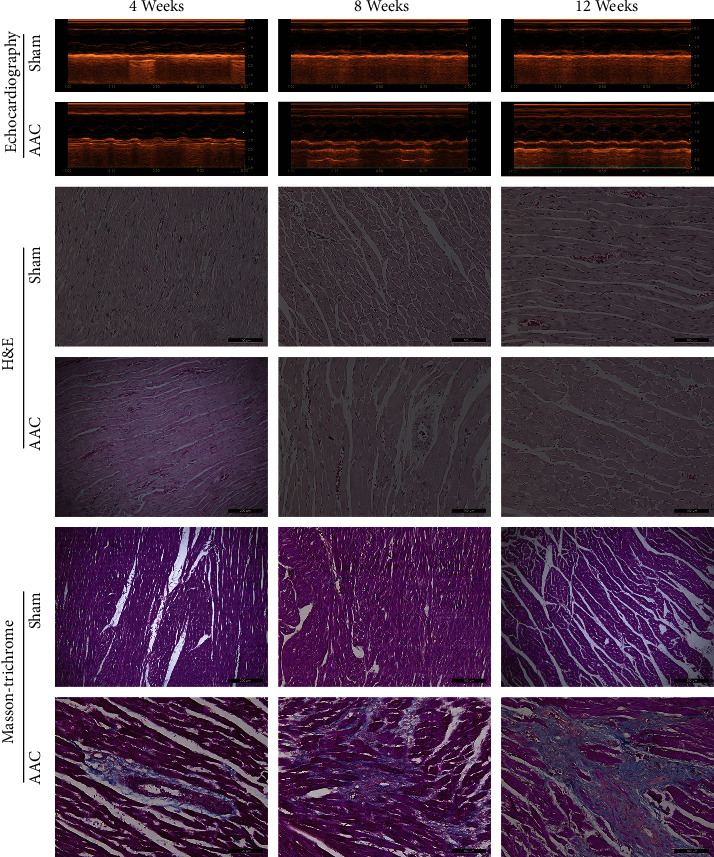
Echocardiography and pathological examination of rat heart. Echocardiography (first and second panels), H&E (third and fourth panels), and Masson's trichrome (fifth and sixth panels) staining of myocardial tissues at 4, 8, and 12 weeks after surgery. Sham: sham operation group; AAC: abdominal aortic constriction; H&E: haematoxylin and eosin. Pathological images were photographed at ×200 magnification.

From the postoperation of AAC to the end of the experiment, there were 6 rats without MH, including 2 rats in the 4 weeks AAC group, 3 rats in the 8 weeks AAC group and 1 rat in the 12 weeks AAC group, 6 rats died, including 1 rat in the 4 weeks AAC group, 1 rat in the 8 weeks AAC group, 2 rats in the 12 weeks sham group, and 3 rats in the 12 weeks AAC group; they were excluded from the study. The remaining 45 rats completed the whole experiment, including 9 rats in the 4 weeks sham group, 7 rats in the 4 weeks AAC group, 9 rats in the 8 weeks sham group and 6 rats in the 8 weeks AAC group and 8 rats in the 12 weeks sham group, and 6 rats in the 12 weeks AAC group. To observe the progression of MH, the hearts of rats were measured by echocardiography. The photo of the M-mode echo-cardiogram indicated (Figure 1) that significant MH was present in the AAC groups of 4 weeks, 8 weeks, and 12 weeks, but the sham group has not changed. The results of the cardiac parameters (Table 2) showed that BW in the AAC group was lower than that in the time-matched group at 4 weeks (*P* < 0.05), 8 weeks (*P* < 0.01), and 12 weeks (*P* < 0.01); HW/BW, LVDs, and LVDd of AAC groups were significantly higher compared with time-matched sham groups (*P* < 0.01, 0.05, 0.05, respectively) at 4 weeks. The HW/BW, IVSd, and LVPWd were significantly higher compared with the time-matched sham groups (*P* < 0.01, *P* < 0.01, and *P* < 0.01, respectively) at 8 weeks and 12 weeks. LVDd in 12 weeks AAC group was higher than that in 12 weeks sham group (*P* < 0.05). No significant differences in LA (4, 8, and 12 weeks), IVSd (4 weeks), LVDs (8, 12 weeks), LVDd (8 weeks), LVPW (4 weeks), LVEF (4, 8, and 12 weeks), and LVFS (4, 8, and 12 weeks) were observed between sham group and AAC group.

### 3.2. Pathological Changes of Myocardial Tissue

H&E and Masson's trichrome staining showed (Figure 1) that significantly increased myocyte size and myocardial interstitial fibrosis was present in the AAC groups at three different time points. Moreover, the MH and myocardial interstitial fibrosis in 8 weeks and 12 weeks AAC groups were severer than in the 4 weeks AAC group. Sham groups had not changed.

### 3.3. Detection of CCK Expression by Immunohistochemical Staining in Myocardial Tissue

**Figure 2:**
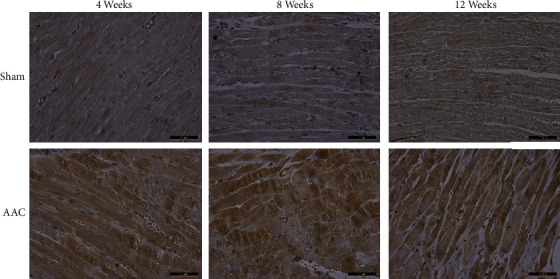
CCK expression levels were detected by immunohistochemical staining (×400 magnification). Sham: sham operation group; AAC: abdominal aortic constriction.

CCK was detected by immune-histochemical staining. CCK antibody used in the experiment was polyclonal antibody. Its method was similar to the previous study about CCK expression in the development of postinfarction heart failure [5]. Expression of CCK was observed in myocardial myofilaments and the nucleus. The expression of CCK was obviously enhanced on the myocardial horizontal stripes, and the expression of CCK was distributed as a “finger pattern” in myocardial tissue under the microscope. The expression of CCK in the nucleus edge was stronger than that in other parts of the nucleus. Staining for CCK was significantly higher in 4 weeks, 8 weeks, and 12 weeks ACC groups compared with those in the corresponding sham groups (Figure 2).

### 3.4. Expression of CCK and CCK mRNA in Myocardial Tissue

**Figure 3:**
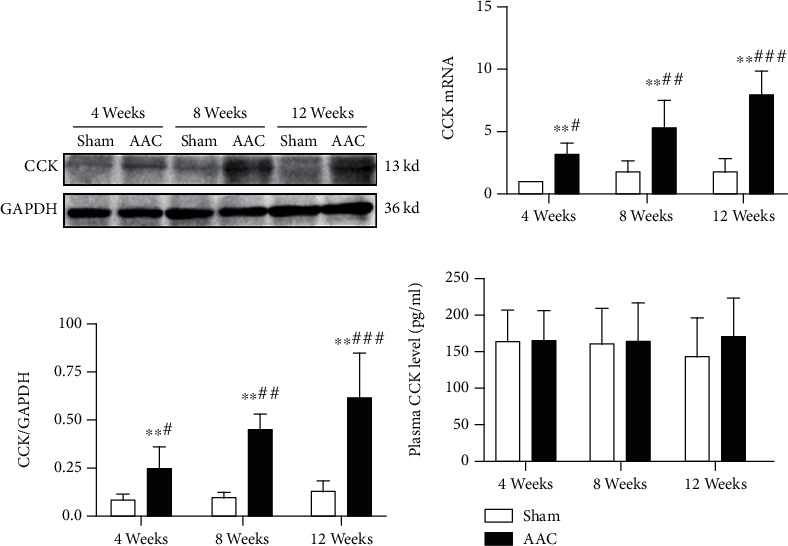
The myocardial tissues of the left ventricular were collected and analyzed by RT-PCR and Western blotting; the plasma samples were collected at the end of experiment and analyzed by ELISA. (a) Analysis of CCK and GAPDH protein expression from myocardial tissues of the left ventricular by Western blotting. (b) Bar graph shows levels of CCK mRNA in myocardial tissue. (c) Bar graph shows relative intensity of CCK to GAPDH in myocardial tissue. (d) The bars indicate the plasma CCK levels. CCK: cholecystokinin; GAPDH: glyceraldehyde-3-phosphate dehydrogenase; ELISA: enzyme-linked immunosorbent assay; sham: sham operation group; AAC: abdominal aortic constriction. ^∗^*P* < 0.05, ^∗∗^*P* < 0.01, compared with sham groups at the same time point; ^#^*P* < 0.01, versus the 8 weeks and 12 weeks. AAC groups; ^##^*P* < 0.01, versus the 4 weeks and 12 weeks AAC groups; ^###^*P* < 0.01, versus the 4 weeks and 8 weeks AAC groups; ^&^*P* < 0.01, versus the 8 weeks and 12 weeks AAC groups.

The relative protein level was measured by WB. CCK antibody used in the experiment was polyclonal antibody. The relative protein level of CCK was significantly higher in 8 weeks and 12 weeks AAC groups than that in the corresponding sham groups (*P* < 0.01 and *P* < 0.05, respectively). Nevertheless, there was not difference between sham and AAC group at 4 weeks. The relative protein level of CCK in the 4 weeks AAC group was less than that in 8 weeks and 12 weeks AAC groups (*P* < 0.05). Quantitative analysis of CCK mRNA showed that CCK mRNA was significantly higher in 4 weeks, 8 weeks, and 12 weeks AAC groups than that in the corresponding sham groups (*P* < 0.01, *P* < 0.01, and *P* < 0.01, respectively). CCK mRNA levels were significantly different from each other in 4 weeks, 8 weeks, and 12 weeks AAC groups (*P* < 0.05) and showed a gradually increasing trend with time (Figure 3).

### 3.5. Measurement of Plasma CCK Levels

Plasma CCK levels were measured by ELISA (Figure 3), which showed that plasma CCK levels were not different between the sham and AAC group at 4, 8, and 12 weeks.

### 3.6. Associations between the Expression of CCK and Left Ventricular Wall Thickness

**Figure 4:**
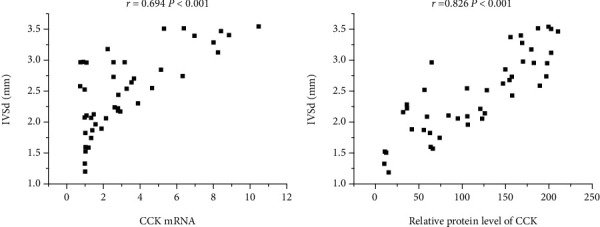
Associations between expression of IVSd and C-CK in model rat. (a) Correlation between IVSd and CCK mRNA (*r* = 0.694, *P* < 0.001); (b) correlation between IVSd and relative protein level of CCK (*r* = 0.826, *P* < 0.001). CCK: cholecystokinin; IVSd: end-diastolic interventricular septal thickness.

**Figure 5:**
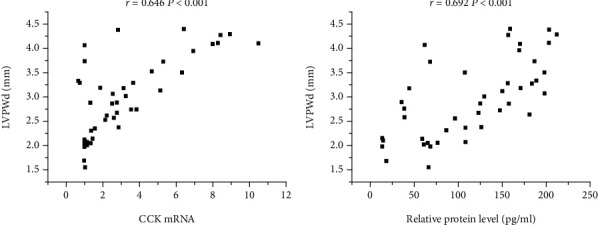
Associations between expression of LVPWd and CCK in model rat. (a) Correlation between LVPWd and CCK mRNA (*r* = 0.646, *P* < 0.001); (b) Correlation between LVPWd and relative protein level of CCK (*r* = 0.692, *P* < 0.001). CCK: cholecystokinin; LVPWd: left ventricular end-diastolic posterior wall thickness.

The left ventricular wall thickness was represented by IVSd and LVPWd measured by echocardiography. The correlation between CCK levels and left ventricular wall thickness was showed in the indicated figure (Figures 4 and 5). IVSd was positively correlated with the CCK mRNA levels (*P* < 0.001) and the relative protein level of CCK (*P* < 0.001) (Figure 4). LVPWd was positively correlated with the CCK mRNA levels (*P* < 0.001) and the relative protein level of CCK (*P* < 0.001) (Figure 5).

## 4. Discussion

Myocardial hypertrophy is induced by heart overload and characterized by increased myocyte size and the incrassate cardiac wall and thought to be an adaptive response to cardiac wall stress resulting from the cardiac pressure afterload and volume overload, including hypertension [12], aortic constriction [13, 14], and valve regurgitation [15, 16]. MH is first described by Cotton in 1914 [17]. With the increasing understanding of MH, we find that if the underlying causes of MH are not removed. It can eventually develop into HF, leading to death from HF. The clinical study also reported MH is associated with morbidity and mortality of patients [18]. As research has progressed, we have found that many factors are related to MH and affect the process of MH, such as sex difference [19], fat diet [20], testosterone [21], angiotensin II [22], isoproterenol [23], and leptin [24].

CCK has various chemical structures, such as CCK-58, CCK-33, CCK-22, CCK-8, and CCK-4, and they work by stimulating the corresponding receptors for CCK [25]. CCK has many biological functions, mainly stimulating the secretion and synthesis of pancreatic enzymes, enhancing the secretion of pancreatic bicarbonate, stimulating gallbladder's contraction and odil sphincter relaxation and the secretion of liver bile, and regulating the movement of the small intestine and colon.

CCK appears to have several effects on the cardiovascular system, although the mechanisms underlying these effects remain unknown [26]. Some studies have shown that CCK is involved in regulating blood pressure and improving heart function [27–29]. In 1998, Zou et al. inserted an end hole PE-50 catheter into heart's left ventricle through the left jugular artery. Left ventricular wall motion modes were recorded during the intravenous administration of CCK-8. The results show the intravenous CCK-8 affected cardiac function [30], and the pretreatment with proglmide (nonselective CCK antagonist; 30 mg/kg; i.p.) causes a further decrease of blood pressure in endotoxic shock, most likely through the CCK AR/BR, which is expressed on cardiac myocytes [31, 32]. Therefore, the relationship between CCK and heart is worthy of further study.

In this study, we produced an MH model of a rat by AAC. The hearts of rats were measured by echocardiography 4, 8, and 12weeks after an operation. We found that IVSd and LVPWd were significantly higher compared with the time-matched sham groups (*P* < 0.01, *P* < 0.01, *P* < 0.01, respectively) at 8 weeks and 12 weeks, but no significant differences in IVSd and LVPW between sham group and AAC group 4 weeks after the operation. The rat hearts were obtained 4, 8, and 12 weeks after the operation and be sliced for H&E and Masson's trichrome staining. H&E and Masson's results showed that significantly increased myocyte size and myocardial interstitial fibrosis were present in the AAC groups at three different time points. Moreover, the MH and myocardial interstitial fibrosis in 8 weeks and 12 weeks AAC groups were severer than in the 4 weeks AAC group; however, sham groups had not changed. Although no difference in ventricular wall thickness (IVSd and LVPWd) was found by echocardiography between AAC and sham 4 weeks after the operation, the size of myocytes of AAC was more than that of sham, and myocardial tissues had obvious fibrosis 4, 8, and 12 weeks after the operation. So, the heart had become hypertrophy 4 weeks after the operation.

CCK in myocardial tissue was detected by IHC. We found that CCK expression in AAC was higher than that in sham 4, 8, and 12 weeks after the operation, and CCK expression was higher in AAC groups compared with sham groups and showed a gradual increase following the change of time in AAC groups. The expression of CCK detected by IHC was consistent with that by WB, but CCK expression detected by WB is not different between the AAC group and sham group at 4 weeks; it may be related to insufficient samples. These results show that MH can affect the expression of CCK.

The known hormonal CCK peptides in the brain and intestine, i.e., ɑ-amidated and tyrosyl-sulfated CCK-58, CCK-33, CCK-22, and CCK-8, were detected only in negligible trace amounts, indicating that only little pro-CCK in the normal heart is processed to ɑ-amidated CCK peptides, which regulate gallbladder emptying, and pancreatic enzyme secretion, and which act as potent neurotransmitters in the central and peripheral nervous systems [33]. On the contrary, we found a considerable amount of CCK expression from the heart in this study. The reason may be related to the antibody used. The antibodies used in IHC and WB were polyclonal antibodies with poor specificity. So, CCK detected by IHC and WB could be pro-CCK. Quantitative analysis of CCK mRNA showed that CCK mRNA was significantly higher in 4 weeks, 8 weeks, and 12 weeks AAC groups than in the corresponding sham groups. CCK mRNA levels were significantly different in 4 weeks, 8 weeks, and 12 weeks AAC groups and showed a gradually increasing trend with time. It indicated that MH could induce the transcription and synthesis of CCK mRNA.

CCK can express in myocardial tissue, but the cause of CCK expression is not clear. A previous report demonstrated that cardiac CCK gene expression in vitro and in vivo is stimulated by isoprenaline [34], which has a strong excitatory effect on *β*-adrenoceptors, and leads to cardiac contraction, an increase in heart rate, and sympathetic excitation; this report reflected that expression of CCK is associated with sympathetic activity. Jens P. Goetze's study found the duodenal.

CCK mRNA did not change 5 h after the rats were injected intraperitoneally with isoprenaline. In contrast, the cardiac CCK mRNA increased by 5-fold [10, 35]. Therefore, isoproterenol can induce high expression of cholecystokinin in myocardial tissues by stimulating *β*-adrenergic receptors. Liu et al.'s study found that the serum norepinephrine level in rats with myocardial hypertrophy was higher than those in control groups 2, 3, and 4 weeks after AAC operation, and it may be related to autonomic excitation induced by AAC operation and pressure overload of the heart [36]. So, MH induced the secretion of catecholamine, which lead to the expression of CCK and transcription of CCK mRNA. However, it was possible that there were other undiscovered causes involved in CCK expression in myocardial tissue.

The clinical study showed the plasma CCK levels were associated with cardiovascular mortality in elderly female patients [8, 37]. Recent animal studies have shown that high expression of CCK can be seen in HF rat myocardial tissue, and the concentration of CCK in plasma of heart failure model rats is higher than that of the non-HF group [5, 22]. In this study, the plasma CCK detected by ELISA is derived from the gastrointestinal tract and the heart. We found that the plasma CCK level was not different between the sham and AAC group at 4, 8, and 12 weeks. The results indicated that little amount of CCK synthesized by the heart could not affect the plasma CCK level during MH. So, the plasma CCK cannot be used as a biomarker for MH.

The correlation analysis indicated that the IVSd and LVPWd were a high-positive correlation with relative protein expression of CCK and CCK mRNA levels in AAC groups. These results suggested that MH can affect the expression of CCK in myocardial tissues, but the mechanism is unclear.

Results from rats with MH provided the basic experimental data and evidence for further studies of CCK in myocardial tissue. The mechanism is not clear, so it deserves further investigation.

## 5. Limitations of the Study

Firstly, we were unable to distinguish CCK from pro-CCK. Secondly, we did not investigate whether CCK was involved in the development of MH. In addition, it had not been identified which subtype of CCK was associated with MH. Further investigations are required to elucidate the specific mechanism of MH affecting CCK expression.

## 6. Conclusion

In conclusion, our study finds that the development of MH induced by AAC can affect the CCK expression of myocardial tissues.

## Figures and Tables

**Table 1:** Sequences of primers used for qPCR.

CCK-F	5′-GTG CTG AGG ACT ACG AAT ACC-3′
CCK-R	5′-GAA ACA TTG CCT TCC CAC-3′
CCKAR-F	5′-CTG GGA TTG TGA TGG TGG TG-3′
CCKAR-R	5′-GCA AGT AAC AGC CAT CAC TAT CC-3′
CCKBR-F	5′-TAA GAA GCG GGT GGT GCG AATG-3′
CCKBR-R	5′-ACA AGC AGA GAC GTA GCT CAG CAAG-3′
GAPDH-F	5′-ATG CCG CCT GGA GAA ACC-3′
GAPDH-R	5′-GCA TCA AAG GTG GAA GAA TGG-3′

F: forward; R: reverse.

**Table 2:** Body weight and echocardiographic evaluation of heart at 4 weeks, 8 weeks, and 12weeks after surgery.

	4 weeks	ACC	8 weeks	ACC	12 weeks	ACC
	Sham		Sham		Sham	
BW (g)	438.11 ± 15.94	418.00 ± 19.21^∗^	551.00 ± 35.04	494.33 ± 23.63^∗∗^	607.00 ± 25.85	512 ± 15.59^∗∗^
HW/BW (mg/g)	3.31 ± 0.05	3.77 ± 0.31^∗∗^	3.95 ± 0.27	4.84 ± 0.52^∗∗^	3.80 ± 0.21	4.54 ± 0.58^∗∗^
LA (mm)	3.48 ± 0.50	3.79 ± 0.59	3.62 ± 0.81	4.13 ± 0.60	4.56 ± 0.80	4.82 ± 0.55
IVSd (mm)	1.88 ± 0.58	2.32 ± 0.26	2.19 ± 0.60	3.10 ± 0.35^∗∗^	2.32 ± 0.34	3.32 ± 0.25^∗∗^
LVDs (mm)	2.16 ± 0.24	2.48 ± 0.25^∗^	2.15 ± 0.40	2.14 ± 0.78	2.37 ± 0.29	2.37 ± 0.47
LVDd (mm)	4.89 ± 0.62	5.52 ± 0.49∗	5.48 ± 0.72	5.54 ± 0.47	6.05 ± 0.38	6.31 ± 0.72^∗^
LVPWd (mm)	2.44 ± 0.84	3.23 ± 0.60	2.31 ± 0.47	3.63 ± 0.57^∗∗^	2.78 ± 0.42	3.97 ± 0.44^∗∗^
LVEF (%)	90.45 ± 1.74	90.14 ± 1.95	93.22 ± 2.59	93.50 ± 5.17	93.63 ± 1.60	93.83 ± 3.31
LVFS (%)	54.89 ± 2.57	54.86 ± 3.93	60.56 ± 6.42	64.67 ± 9.75	60.75 ± 4.77	61.83 ± 8.89

Data was presented as the mean ± standard deviation (SD). Sham: sham operation group; AAC: aortic artery constriction; BW: body weight; HW: heart weight; LA: left atrial diameter; IVSd: end-diastolic interventricular septal thickness; LVDd: left ventricular end-diastolic diameter; LVDs: left ventricular end-systolic diameter; LVPWd: left ventricular end-diastolic posterior wall thickness; LVEF: left ventricular ejection fraction; LVFS: left ventricular short-axis f-ractional shortening. ^∗^*P* < 0.05, ^∗∗^*P* < 0.01, compared with the time-matched sham groups.

## Data Availability

The datasets used and/or analyzed during the current study are available from the corresponding author on reasonable request.

## Abbreviations

CCK: Cholecystokinin
ACC: Abdominal aortic constriction
MH: Myocardial hypertrophy
HF: Heart failure
HW: Heart weight
IVSd: Interventricular septal thickness at end-diastole
LVDd: Left ventricular end-diastolic dimension
LVDs: Left ventricular end-systolic dimension
LVPWd: Left ventricular posterior wall thickness at end-diastole
LVEF: Left ventricular ejection fraction
LVFS: Left ventricular factional shorting
IHC: Immunohistochemistry
WB: Western blotting
qPCR: Quantitative real-time polymerase chain reaction.
